# Social network correlates of risky sexual behavior among adolescents in Bahir Dar and Mecha Districts, North West Ethiopia: an institution-based study

**DOI:** 10.1186/s12978-018-0505-8

**Published:** 2018-04-11

**Authors:** Kerebih Asrese, Alemtsehay Mekonnen

**Affiliations:** 1 Faculty of Social Sciences, Bahir Dar University, Bahir Dar, Ethiopia; 2 College of Medicine and Health Sciences, Reproductive Health Department, Bahir Dar University, Bahir Dar, Ethiopia

**Keywords:** Adolescents, Risky sexual behavior, Social network

## Abstract

**Background:**

Behaviors established during adolescence such as risky sexual behaviors have negative effects on future health and well-being. Extant literature indicated that individual attributes such as peer pressure and substance use have impacts on healthy development of young peoples’ sexual behavior. The patterns of relationships (social network structure) and the social network content (members’ norm regarding sexual practice) established by adolescents’ network on adolescents’ risky sexual behaviors are not well investigated.

**Methods:**

This cross-sectional study assessed the roles of social networks on sexual behavior of high school adolescents in Bahir Dar and Mecha district, North West Ethiopia. Data were collected from 806 high school adolescents using a pretested anonymously self administered questionnaire. Hierarchical logistic regression model was used for analysis.

**Results:**

The results indicated that more than 13% had risky sexual behavior. Taking social networks into account improved the explanation of risky sexual behavior over individual attributes. Adolescents embedded within increasing sexual practice approving norm (AOR 1.61; 95%CI: 1.04 – 2.50), increasing network tie strength (AOR 1.12; 95% CI: 1.06 – 1.19), and homogeneous networks (AOR 1.58; 95% CI: .98 – 2.55) were more likely to had risky sexual behavior. Engaging within increasing number of sexuality discussion networks was found protective of risky sexual behavior (AOR .84; 95% CI: .72 – .97).

**Conclusion:**

Social networks better predict adolescent’s risky sexual behavior than individual attributes. The findings indicated the circumstances or contexts that social networks exert risks or protective effects on adolescents’ sexual behavior. Programs designed to reduce school adolescents’ sexual risk behavior should consider their patterns of social relationships.

## Plain English summary

Behaviors established during adolescent might negatively affect the healthy development of young peoples’ sexual behavior. The effects of adolescents’ patterns of relationships- social networks and the content of such relationships on their risky sexual behavior are not well investigated.

Respondents were asked their background characteristics, sexual behavior, the people with whom they discussed sexual matters, the strength of the relationship they have with these people, the homogeneity/heterogeneity of the relationship (whether the network members are kins or non-kins), and the opinion of these people about sexual practice.

Eight hundred six respondents with age rage 15 – 23 years participated in the study; more than 13% had risky sexual behavior. Taking social networks into account improved the explanation of risky sexual behavior over individual attributes. Adolescents embedded within increasing sexual practice approving norm networks (AOR 1.61; 95%CI: 1.04 – 2.50), increasing network tie strength (AOR 1.12; 95% CI: 1.06 – 1.19), and homogeneous networks (AOR 1.58; 95% CI: .98 – 2.55) were more likely to had risky sexual behavior. Engaging within increasing number of sexuality discussion network members was found protective of risky sexual behavior (AOR .84; 95% CI: .72 – .97).

In sum; social network variables predicted adolescent’s risky sexual behavior more so than individual attributes. The findings indicated that patterns of relationships (network structure) and the network content (network members’ sexual norm) exert risks or protective effects on adolescents’ sexual behavior. Programs designed to reduce school adolescents’ sexual risk behavior should consider their patterns of social relationships.

## Background

Adolescence is the time of transition from childhood to adulthood during which young people experience changes following puberty. It is one of the stages of development characterized as a period of change, vulnerability and opportunity [1]. During adolescence, youth question their identity, seek to establish relationships outside the family environment, begin to understand and experience their sexuality, and begin to prepare themselves to enter the labor market [1]. Engagement in risky behaviors such as substance use, violence perpetration, and unsafe sexual practices are common during adolescence and are significant public health concerns [2–4].

Adolescents in Ethiopia (age range 15 – 24) comprised of 20.6% of the 73.4 million people [5]. This portion of the population is experiencing adverse health outcomes owing to their sexual behaviors [6]. Significant proportions adolescents in Ethiopia engaged in risky sexual behavior such as early sexual initiation, multiple partner sexual relationships, unprotected sex, and sex in ex-change of money [6, 7]. Thus, preventable sexual behavior morbidities including sexually transmitted diseases, HIV/AIDS and other reproductive health problems such as unintended pregnancy and unsafe abortion are the greatest threats to their well-being [8–10].

To date, efforts to explain the risky sexual behavior among the Ethiopian adolescents have primarily focused on analyzing the individuals’ attributes such as substance use, alcohol consumption, and exposure to erotic video films [11, 12]. Existing literature documented that sexual behavior is learned and is reinforced through network members’ behavioral examples, the normative environments they create, and the pressures they exert, as well as individuals’ perceptions of the behavior [13]. Thus, to better understand the risky sexual behavior of adolescents, it is important to explore the social contexts in which sexual behaviors among adolescents are developed and shaped [14, 15].

The social network literature on sexual behavior posited that social networks influence sexual behavior of network members by serving as sources of information about specific sexual behaviors as well as about potential sex partners and often introduce individuals to their future sex partners (15). Social network members often also exercise informal control over sexual behavior by creating restrictive normative contexts, by discouraging certain kinds of behaviors, and by exercising other forms of informal social control [14, 16, 17]. Previous studies reported that adolescents who perceive their friends are engaged in sexual practices are more likely to adopt those same behaviors [8, 13].

Social network analyses offer a way to move the analytic focus one step above the individual factors to consider the patterns of relationships through which individuals are embedded in the social environment [18]. This study aims to characterize the associations of social network characteristics and sexual risk behaviors of adolescents. We propose a new perspective which focuses on the structural characteristics of the social network (network size, homogeneity, and network tie strength) and the social network content (network members’ norm regarding sexual practice) those results from adolescents’ network and the influence on adolescents’ risky sexual behavior. We used ego-centered networks data that consisted of a focal actor, termed ego and a set of alters who have specific ties to the focal ego actor. This approach is a widely used instrument to study the relational environment surrounding individuals [19, 20]. In this study, the focal ego actors are the adolescent who completed the survey questionnaire and alters are the network members named by egos who have relations with the ego.

Understanding such social and contextual factors that influence sexual behaviors of adolescent is vital in designing and implementing tailored sexual risk reduction interventions. Building on the existing literature, the study widened the scope of investigation and assessed whether social network characteristics or individual behavioral patterns were important in predicting adolescent risky sexual behaviors. Thus, we hypothesized that adolescent social networks better explain adolescents risky sexual behavior than their individual attributes. We also predicted that adolescents embedded within higher sexual practice approving norm network members had risky sexual behavior.

## Methods

### Study setting and participants of the study

This cross-sectional study was conducted among high school adolescents in Bahir Dar city administration and in Mecha District, West Gojjam Zone, Amhara Region from November12-28/2016. There are eight high schools in Bahir Dar and six high schools in Mecha District. Two schools providing 9 – 10 grades education were randomly selected from each site and sections were selected randomly from each school. The study population was randomly selected from the four high schools proportionate to the student population size.

The sample size for the study was determined using single population proportion formula considering 50% proportion, 95% confidence interval with 5% margin of error, design effect (2) and 10% non response rate. The final sample size was 844 adolescents.

### Study procedure

The study received ethical approval from Ethical Review Committee of Bahir Dar University. We initially developed the survey questionnaire in English and then translated into Amharic to ease understanding. Prior to the study a pretest was conducted among 35 students in a school not selected for the study and the necessary adjustment in language and content was done.

One week before data collection, we communicated principals in the selected schools with formal letters and we selected students who would participate in the study. For selected participants below age 18, we obtained consent from parents/guardians through letters wrote by the principals in the selected schools and verbal assent of individual participants was obtained after being fully informed of the study purpose and procedures. From study participants aged 18 and above, we obtained verbal consent. We ensured confidentiality by removing all personal identities from the questionnaire. At each school, the questionnaires were self administered in a free classroom in the opposite shift without the presence of teachers. The principal investigators and two research assistants informed the participants carefully about the study and were available throughout the administration of the questionnaires to answer questions from individual students.

### Variables and measurements

#### Background variables

Background variables included age, sex, residence, living arrangement with parents/guardians, and substance use. These variables are reported influence adolescents sexual behavior [11, 12, 21].

*Living arrangement*: respondents were asked with whom they are living during the last 12 months before the survey. The responses alternatives were with both biological parents, with single parents (with either mother or father), and with others (relatives, friends, guardians).

*Substance use:* We collected information regarding alcohol drinking and chewing *khat* to measure adolescents’ substance use behavior. Alcohol consumption was measured using the following item: Do you ever drink any form of alcohol during the last 12 months? Response alternatives were never, sometimes (about once a month), and quite regular (every week). Adolescents’ *khat* use status was also measured by asking the following item: Do you ever chew *khat* during the last 12 months? The responses were never, sometimes (about once a month), and quite regular (every week).

### Network characteristics

Four items - network size, network tie strength, network homogeneity, and network members’ sexual norm (approval or disapproval of sexual practice) were included in the questionnaire to measure respondents’ network characteristics [22].

#### Network size

As stated above, the ego is the respondent who directly participated in the survey research, while the alters are people (named by the respondent using the name generator question). Network size is the number of alters named by the ego, who have discussed about sexuality (sexual partner, sexual practice, condom use) during the last 12 months preceding the survey.

Higher number of alters named by the ego indicates higher number of network size.

#### Network tie strength

Using the name interpreter question, respondents were asked to rate how close they felt to each member of the network mentioned using a three point scale (distant = 1, close = 2, and very close = 3). Respondents would rate their relationship with the network member very close when they had closest contact, met more often, discussed secrets, and supported each other as they wish. They would rate the relationship as close when they met occasionally and they felt that they were friends. They would rate the relationship as distant when the contact happened if necessary. Such approach helps to define one’s closest network members [13]. In addition, a visual display of concentric circle that has three circles was used for illustration to help respondents locate their network members in one of the circles. For example, they would locate the very close network member in the inner most circle and distant network members in the outer most circle. The total score of the measure was then calculated, with higher score indicating embedded within strong tie network.

#### Network homogeneity

Using the name interpreter question, respondents were asked whether the network members mentioned in the name generator question were kin or non-kin (non-kin = 0, kin = 1). The sum of valid responses divided by the number of valid response was used to compute the summary measure of network homogeneity of the respondents. The value ranges from 0 – 1with higher score indicating embedded within homogeneous tie network.

#### Network members’ sexual norm

Respondents were asked about the opinion/approval of their network members about sex and sexual practice. The response options were dichotomous (0 = not approved, 1 = approved). Then the summary measure of sexual norm score was obtained by dividing the sum of valid responses by the number of valid responses. The value ranges from 0 to 1, with higher score indicating embedded within networks that approve sexual practice.

#### Sexual behavior

We adapted three items from a previous study to measure sexual behavior of adolescents [8]. We asked whether the respondents had engaged in sexual intercourse during the past 12 months, the number of sexual partners during the past 12 months, and if the respondents consistently used condom during sexual intercourse. Those having more than one partner or not consistently used condom were considered to be in risky sexual behavior. Then, those engaged in risky sexual behavior were coded “1” and the remaining “0”.

### Data analyses

All returned questionnaires were checked for completeness and consistency of responses manually. After cleaning, the items were coded and entered, in to SPSS for Windows versions 23 for analyses.

The dependent variable in this analysis was sexual behavior (coded 1 = risky and otherwise 0). Independent variables included both individual-level variables and network-level variables (network size, network tie strength, network homogeneity, and network members’ sexual norm). Thus, multivariate logistic regression models were used to assess the relationship between independent variables and outcome variable. Nested models were employed to show the unique contribution of social network variables to the understanding of outcome variable. The first was a reduced model including only individual-level characteristics. The second model added network variables to the individual model. Since the first model was nested in models2, we used a likelihood ratio statistic (G^2^) to test whether the addition of network variables significantly improved the fit of the model [23, 24].

## Results

### Background and network characteristics of respondents

From 844 eligible adolescents, 806 (95.5%) fully responded to the self administered questionnaire. Respondents’ age ranges from 15 to 23 years with mean age of 18.72 years. As indicated in Table 1, 72.3% of the participants were males and 64% are rural residents (walk every day from rural sites to attend their education). More than 8 in 10 participants (84.4%) are living with both biological parents, about 1 in 10 participants (11%) are living with single parents, and 4.6% are living with either with relatives, guardians, or alone. More than 4 in 10 participants (40.7%) drink alcohol sometimes (about once a month) and 3.5% drink alcohol quite regularly (every week). More than 1 in 10 adolescents (14.6%) chew khat sometimes (about once a month) and 2.5% chew khat quite regularly (every week).

**Table 1:** Background and network characteristics of participants (*N* = 806)

Variables	N (%)
Age^a^	18.72(1.09)
sex	
Male	583(72.3)
Female	223(23.7)
Residence	
Rural	516(64)
Urban	290(36)
Living arrangement	
With both parents	680(84.4)
With single parents	89(11)
With others	37(4.6)
Drinking alcohol	
Never	450(55.8)
Some times	328(40.7)
Quite regular	28(3.5)
Chewing khat	
Never	668)82.9)
Some times	118(14.6)
Quite regular	20(2.5)
Perceived family connectedness^a^	11.69(2.41)
Network size^a^	8.69(3.91)
Network tie strength^a^	17.68(9.19)
Network homogeneity^a^	.44(.39)
Network members’ sexual norm^a^	.32(.30)

^a^mean(sd)

On average, participants have identified names of 8.69 network members (sd = 3.91) with whom they discussed about sexuality. The average network tie strength score was 17.68 (sd = 9.19), average network homogeneity score was .44 (sd = .39), and the average sex/sexual practice approval norm score was .32(sd = .30) (Table 1).

### Sexual Behavior of Adolescents

Of all respondents, 221(27.4%) adolescents reported ever had sexual intercourse with mean age at first sexual intercourse by age 16.2. Among those sexually active adolescents, about 60% had sexual intercourse during the past 12 months preceding the survey; nearly half (48.5%) had multiple sexual partner and 66% did not use condom consistently during intercourse. Among all adolescents who participated in the study, 13.2% had risky sexual behavior (Table 2).

**Table 2:** Sexual behavior among adolescents in Bahir Dar and Mecha districts, Amhara Region (*N* = 806)

variables	No (%)
Ever had sex	
Yes	221(27.4%)
No	585(72.6%)
Had sex in the past 12 months	
Yes	132(59.7%)
No	89(40.3%)
Age at first sex	
14 -15 years	97(43.9%)
16 - 17 years	78(35.3%)
> = 18 years	46(20.8%)
Number of sexual partner in the past 12 months	
One	68(51.5%)
Two and above	64(48.5%)
Had protected sex in the past 12 months	
Yes	45(34.1%)
No	87(65.9%)
Sexual behavior	
Healthy	700(86.8%)
Risky	106(13.2%)

### Social network and sexual behavior

Table 3 presents the results of multivariate logistic analyses of the association between sexual behavior in the last year and various individual level and network characteristics of respondents. Model 1 included only individual-level attributes of adolescents. As revealed by Model 1, compared to males, females were 2.48 (95% CI, 1.56 – 3.93; *p* < 0.05) times more likely to have risky sexual behavior in the last year. Compared to adolescents living with both biological parents, adolescents living with single parents were 5.13 (95% CI, 3.05 – 8.72; *p* < 0.01) times more likely to have risky sexual behavior in the last year. Adolescents who drank alcohol sometimes (OR, 3.37; 95% CI, 1.45 – 3.87; p < 0.01) and quite often (OR, 3.60; 95% CI, 1.30 – 9.98; *p* < 0.05) were more likely to have risky sexual behavior than adolescents who did not drink alcohol. Compared to adolescents who did not chew *khat*, those chewing khat sometimes were 1.85 (95% CI, 1.06 – 3.23; p < 0.05) times more likely to have risky sexual behavior.

**Table 3:** Hierarchical Logistic Regression analysis of adolescents’ individual attributes and their social network variables predicting risky sexual behavior (*N* = 806)

variables	Odds ratio (95% confidence interval)	
	Model I	Model II
Age of respondents	1.04(.84 – 1.30)	1.35(1.05 – 1.74)*
Sex of respondents		
Male®	1	1
Female	2.48(1.56 – 3.93)*	2.50 (1.50 – 4.18)**
Residence		
Rural®	1	1
Urban	.872(.55 – 1.38)	1.42(.84 – 2.40)
Living arrangement		
With both parents®	1	1
With single parents	5.13(3.05 – 8.72)**	5.00(2.82 – 8.87)**
With others	1.39(.51 – 3.79)	1.75(.62 – 4.95)
Drinking alcohol		
Never ®	1	1
Sometimes	3.37(1.45 – 3.87)**	2.42(1.43 – 4.13)**
Quite often	3.60(1.30 – 9.98)*	3.68(1.27 – 10.70)*
Chewing khat		
Never ®	1	1
Sometimes	1.85(1.06 – 3.23)*	1.88(1.04 – 3.40)*
Quite often	2.48(.78 – 7.72)	2.25(.66 – 6.59)
Network size	–	.84 (.72 – .97)*
Network members’ sexual norm	–	1.61(1.04 – 2.50)*
Network tie strength	–	1.12(1.06 – 1.19)**
Network homogeneity	–	1.58(.98 – 2.55)*
Model G^2^ (−2log likelihood)	316.478	228.702
Degree of freedom	9	13
Changed Chi-square	90.985**	133.532**

Note: *p < .05; **p < .01; ® = reference category

Model 2 added four network variables to Model 1 (network size, network members’ sexual norm, network tie strength, and network homogeneity). The results showed that some of the formerly significant variables (living with single parents, drinking alcohol, being female, and chewing *khat* sometimes) remained significant. Other individual level variable which was not significant in model I (age) became significant in model II. With each one year increase in age (range 15 – 23 years), respondents were 1.35times more likely to have risky sexual behavior (95% CI, 1.05 – 1.74; *p* < 0.05).

As illustrated in model 2, network variables were significantly associated with risky sexual behavior of adolescents. With each one member increase in network size with whom ego discussed about sexuality (range 2 – 13) adolescents were .84 times less likely to have risky sexual behavior (95% CI, .72 - .97; p < 0.05). With each point increase in network homogeneity score (range 0 – 1), adolescents were 1.58 times more likely to have risky sexual behavior (95% CI, .98 – 2.55; *p* < 0.01) and with each point increase in network members’ sexual practice approval norm score (range 0 – 1), adolescents were 1.61times more likely to have risky sexual behavior (95% CI, 1.04 – 2.50; *p* < 0.01). And with each one point increase in network members tie strength measure, respondents were 1.12 times more likely to have risky sexual behavior (95% CI, 1.06 – 1.19; p < 0.01).

As revealed in Table 3, model II had smaller G^2^ value than model I (228.702 vs. 316.478). The Chi square analysis of changes in Model G^2^ values revealed that the inclusion of network variables significantly improved the goodness-of-fit of Model II as compared to Model I (χ2 = 133.532, df = 13, *N* = 806, *P* < 0.001). The results indicated that there is statistically significant improvement in predicting risky sexual behavior of adolescents with the network variables after controlling individual level variables. Thus, the results revealed that adolescents’ social network variables better predict their sexual behavior than their individual attributes.

## Discussion

This study assessed social networks and sexual behavior in a sample of adolescents in Bahir Dar city and Mecha district, North West Ethiopia. In this sample of adolescents, more than 27% of adolescents in grades 9 and 10 initiated sexual activity. Of all adolescents who participated in the study, 13.2% had risky sexual behavior.

As we hypothesized, the analysis of the factors for risky sexual behavior revealed that social network variables predict adolescents’ risky sexual behavior more so than individual attributes. As compared to an individual-level model, the inclusion of social network variables significantly improved the fit of the models predicting risky sexual behavior. The results demonstrated that risky sexual behavior was better predicted with the addition of network variables than adolescents’ individual attributes alone. The results corroborate previous studies [8, 11, 25] that have reported the importance of the social context for adolescents’ sexual behavior. In a recent study, Cherie& & Berhane reported that perception of peers’ involvement in risky sexual practice was strongly associated with risky sexual behavior of adolescents [8]. Therefore, interventions that will bring peer influence for healthy sexual behavior development should be sought and strengthened at schools.

This study also anticipated that adolescents embedded within higher sexual practice approving norm network members had risky sexual behavior. The results pointed out that embedding within higher sexual practice approving norm networks was found more likely to lead to engagement in risky sexual behavior. This finding is consistent with previous studies that peer pressure was one of the significant factors for engagement in risky sexual behavior among adolescents [11, 26, 27]. Social interaction within social networks is thought to influence behavior through social learning and social influence. Social learning emphasizes the role of interactions and information in reducing uncertainties associated with new behaviors and practices. Social influence extends beyond social learning. It implies that social networks reinforce or alter norms by providing examples of behavior that may then be considered and copied by others [28]. In this study, it is not clear whether adolescents engage in risky sexual behavior to conform to an existing social norm (social influence) or whether those adolescents who engage in risky sexual behavior are drawn to other adolescents who also engage in risky sexual behavior (social learning). Regardless of the direction, the findings highlight the importance of social networks in influencing adolescents’ sexual behavior. Thus, programs designed to reduce risky sexual behavior may focus on adolescents’ perception of normative behavior.

Compared to previous studies, this study has two important contributions: applying a social network approach to investigate risky sexual behavior among adolescents and demonstrating the importance of measuring the ego-alter ties and identifying specific social network structures and network content (norm) that are risk or protective for adolescents’ risky sexual behavior. Though previous studies [8, 25] had identified social context as ‘umbrella’ in influencing adolescents’ sexual behavior, a social network approach that considered the patterns of relationships (network structures) and the network contents (normative behavior of members) were not investigated. This study extends our understanding of the roles that social networks shape the adolescents’ sexual behavior through its patterns of relationships (structures) and network content. In addition, this study measured specific types of structures and or content that a social network may comprised of and demonstrated the relative importance of these structural variables and or content in influencing adolescents’ risky sexual behavior.

Interestingly, the network structural variables included in the study i.e., network size, network tie strength, and network homogeneity had significant relationship with egos’ risky sexual behavior. We found that an increase in the size of sexuality discussion network was protective of risky sexual behavior. This finding is in contrast to a study done in Ghana that reported having more friends increased the odds of multiple sexual partners among young respondents [28]. Network tie strength that indicates egos’ intimacy of the relationship with network members was found significantly associated with egos’ risky sexual behavior. This finding also contradicts earlier study in U.S. that reported best friends were protective of engaging in risky sexual behavior [29]. These inconsistencies in findings may be attributed to the different cultural contexts of the adolescent population.

Our findings further revealed that adolescents embedded within higher homogeneous network members (many of the members are kin) were more likely engaged in risky sexual behavior. Such pattern of relationship limits adolescents’ social world [30] that has powerful implications for information to learn different social norm, thus behave the sexual norms of their fellow members.

This study has several limitations that should be acknowledged. Since adolescents were asked to recall retrospectively their network members and their roles for their sexual behavior, there may be memory lapses that affect the accuracy of recall. Adolescents may also report inaccurate data or they may give responses which they believed to be expected or acceptable, thus lead to underestimation of risky sexual behaviors. To minimize this bias, efforts were made to provide a safe environment to complete the questionnaire and omitting all identifying variables from the questionnaire to ensure anonymity.

We collected data only from adolescents who participated in the study. The lack of data from other informants (e.g., family members, other network members) is another weakness of the study. In addition, the cross-sectional nature of the study limited the interpretation of the findings in terms off cause-effect relationships. There are also many factors this study did not assess, including community influences (neighborhood) and intrapersonal influences (e.g., religious affiliations, spirituality). Future research may attempt to address these factors into consideration to predict risky sexual behavior among adolescents. Studies on factors contributing to positive adolescent development are also needed to assess the assets in and outside adolescents so that interventions and programs which can foster positive behavior can be developed and implemented.

## Conclusion

This study assessed the sexual behavior of adolescents and investigated whether adolescents’ social networks or individual characteristics were important in explaining their sexual behavior in Bahir Dar and Mecha district, North West Ethiopia. Sex, residence, living arrangement, and age were the individual level variables assessed in the study. Network size, network tie strength, network members’ sex/sexual practice norm, and network homogeneity were the network variables considered in the study.

The findings indicated that more than 27% of adolescents in grades 9 and 10 initiated sexual activity. More than 13% had risky sexual behavior. The hierarchical logistic regression results revealed that adolescents’ social networks better predict their sexual behavior than their individual attributes alone. The study also indicated the circumstances or contexts that social networks exert risks or protective effect on adolescents’ sexual behavior. Adolescents embedded within increasing sex/sexual practice approving norm (network content) as well as strong tie networks and those embedded within homogeneous networks were more likely to had risky sexual behavior in the last 12 months. On the other hand, increasing network size of sexual issue discussing networks was protective of risky sexual practices in the last 12 months. These findings are suggestive that programs that are intended to promote healthy sexual behavior of adolescents may use the patterns of adolescents’ relationships (network structures) and network content (normative behavior of the members) as a resource to protect them from risky sexual behavior.
